# Characterization of expression patterns and dynamic relocation of Notch proteins during acrosome reaction of bull spermatozoa

**DOI:** 10.1038/s41598-024-65950-0

**Published:** 2024-06-28

**Authors:** Patrícia Diniz, Inês Leites, Mariana R. Batista, Ana Catarina Torres, Luísa Mateus, Luís Lopes-da-Costa, Elisabete Silva

**Affiliations:** 1 Reproduction & Development Lab, CIISA – Centre for Interdisciplinary Research in Animal Health, Faculty of Veterinary Medicine, University of Lisbon, Lisbon, Portugal; 2 Associate Laboratory for Animal and Veterinary Sciences (AL4AnimalS), Lisbon, Portugal; 3 Faculty of Veterinary Medicine, Lusófona University - Lisbon University Center, Lisbon, Portugal

**Keywords:** Notch, Sperm, Capacitation, Acrosome reaction, Bull, Extracellular signalling molecules, Checkpoint signalling

## Abstract

Notch is a conserved cell-signaling pathway involved in spermatogenesis regulation. This study firstly evaluated the presence, localization patterns, acquisition origin and relation to acrosome reaction of Notch proteins in bull sperm. Western Blot analysis detected all Notch proteins in ejaculated bull sperm, and immunostaining described their specific sperm localization. Recovery of sperm from different segments showed that Notch proteins have testicular origin (NOTCH1, NOTCH2, DLL4), are sequentially acquired during sperm maturation along epididymal transit (NOTCH3, DLL3, JAGGED1-2), or post-ejaculation (DLL1, NOTCH4). Testis NOTCH2 is ubiquitously expressed in all germ-cell lines, whereas DLL4 is expressed in round and elongated spermatids during the Golgi, Cap, Acrosome and Maturation phases. In vitro spontaneous and induced sperm acrosome reaction induce consistent sperm regional relocation of NOTCH2, DLL4 and JAGGED1, and these relocation patterns are significantly associated to sperm acrosome status. NOTCH2 and JAGGED1 are relocated from the head apical to the post-equatorial regions, whereas DLL4 is lost along with the acrosome, evidencing that sperm spatial redistribution of NOTCH2 and JAGGED1 is linked to acrosome reaction onset, whereas DLL4 loss is linked to AR completion. Overall, results prompt for a relevant Notch role in bull sperm acrosome testicular development, epididymal maturation and acrosome reaction.

## Introduction

In mammals, following spermatogenesis, sperm cells undergo post-testicular maturation in the epididymis to acquire forward motility and fertilizing ability^1,2^. This maturation involves extensive remodeling of the sperm membrane^3^, with relocation of surface proteins^4,5^, and being other proteins modified, masked, or replaced by proteins of epididymal origin^6,7^. Epididymosomes, extracellular vesicles released from the epididymal epithelium, are a major vehicle of epididymal protein transfer to maturing sperm^8^. Additionally, during urethral transit and in the female reproductive tract, sperm cells come into contact with seminal plasma and utero-oviductal proteins, which lead to sperm capacitation^9^. Sperm acrosome reaction, a late capacitation pre-requisite for sperm-oocyte fusion, is a Ca^2+^-dependent exocytotic event involving the fusion of sperm acrosomal and plasma membranes^10,11^. Following acrosome exocytosis, the post-acrosomal segments remain intact, exposing the site for sperm-egg fusion^12^. Acrosome exocytosis is also accompanied by a translocation of proteins to the equatorial segment. In particular, the plasma membrane of the equatorial segment becomes denser with protein complexes that are redistributed from the apical region^13,14^. These changes in the equatorial segment of the sperm are essential for the sperm to penetrate the egg and complete fertilization.

The Notch signaling pathway is an evolutionarily conserved mechanism that regulates a broad spectrum of cell fates and developmental processes^8,15^. In mammals, four receptors (NOTCH1-4) and five ligands (JAGGED1-2; *Delta-like*1, 3 and 4) are identified^16^. Notch canonical signaling is activated by biding of a membrane ligand with the transmembrane receptor of a neighboring cell. Notch non-canonical signaling is either dependent or independent of a ligand, acting through cross-talking with Wnt/β-Catenin or other signaling pathways^17,18^. Notch signaling components were identified in testis germ and Sertoli cells in several mammalian species^19–25^, evidencing a regulatory role in the spermatogenic cycle. The team’s previous studies in a mouse model demonstrated the presence of Notch proteins in testis germ, Sertoli and Leydig cells^25^, the epithelium of the epididymis and the *vas deferens*, within epididymosomes epididymal sperm cells^26^. In vivo Notch signaling blocking resulted in apoptosis of germ cells, generation of abnormal spermatozoa, and a significant decrease in the proportion of sperm with progressive forward motility^27^. Although Notch involvement in spermatogenesis has been subject of several studies, the relationship between Notch proteins and sperm capacitation and acrosome reaction remains unknown. Also, studies mainly addressed lab rodents and humans^19–25^, whereas the picture in farm species, namely in bull sperm, is largely fragmental. Novel knowledge on sperm capacitation may pave the way to improvement of assisted reproductive techniques (ART).

The objective of this study was to evaluate the dynamic localization of Notch proteins in bull sperm cells, from their germ cell origin during spermatogenesis, to their epididymal and *vas deferens* transit, up to ejaculated cells. Additionally, the study evaluated the localization and relocation of Notch proteins during acrosome reaction.

## Results

### Presence and localization patterns of Notch components in bull ejaculated sperm cells

**Figure 1:**
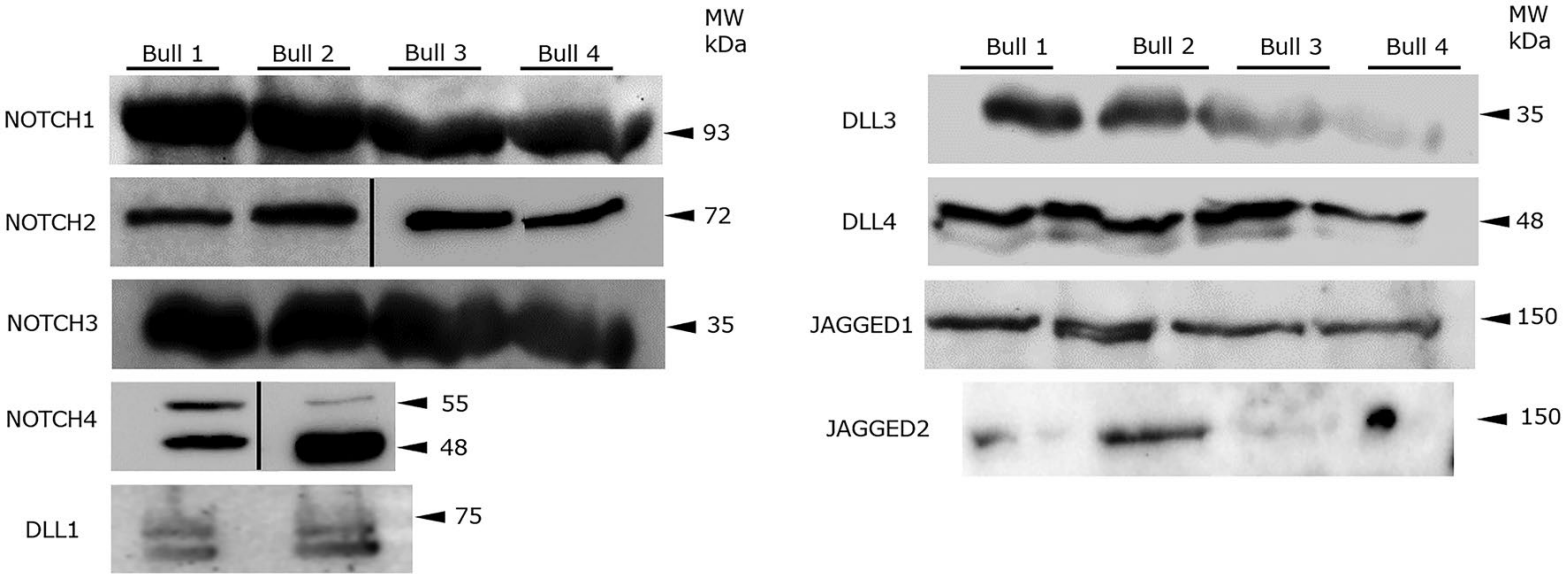
Western blot analysis of Notch proteins in bull ejaculated sperm cells. Full-length blots are available in Supplementary Figure S1 and Supplementary Figure S2. NOTCH1 was detected at approximately 93 and 235 kDa, corresponding to the active form and full-length protein, respectively. NOTCH2 was detected at approximately 72 kDa, corresponding to NOTCH2 intracellular domain. NOTCH3 was detected at approximately 35 kDa; NOTCH4 at 55 and 48 kDa; DLL1 full-length protein at around 78 kDa; DLL3 at 35 kDa; DLL4 at 48 kDa; full-length JAGGED1 protein and JAGGED2 at around 150 kDa. The vertical lines in NOTCH2 and NOTCH4 blots indicate lanes from different gels.

**Figure 2:**
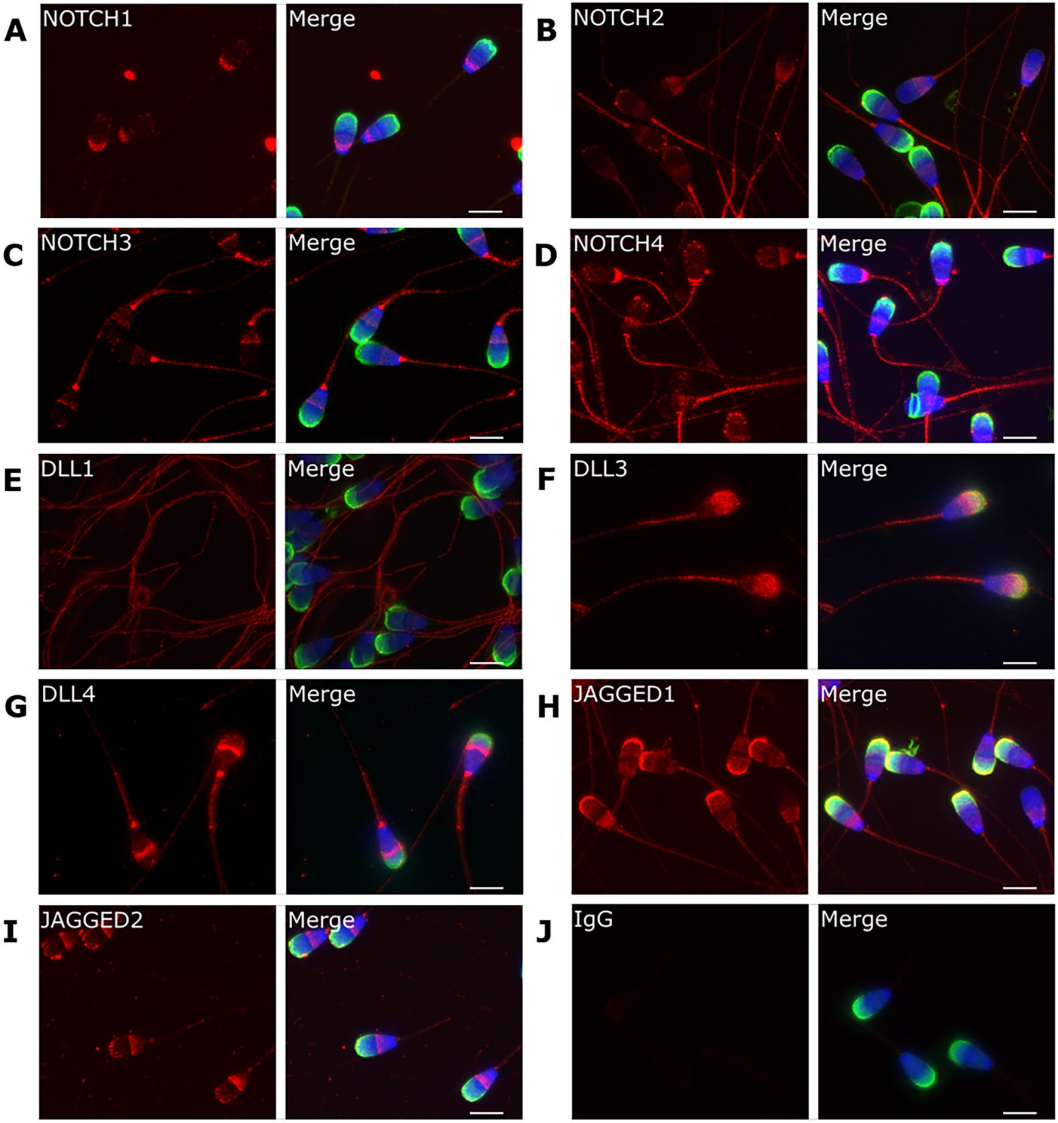
Representative images of Notch proteins immunostaining in bull sperm cells, showing the localization patterns of NOTCH1 (**A**), NOTCH2 (**B**), NOTCH3 (**C**), NOTCH4 (**D**), DLL1 (**E**), DLL3 (**F**), DLL4 (**G**), JAGGED1 (**H**), JAGGED2 (**I**). Negative control with IgG (J). Secondary antibody—Alexa 594® (red); acrosome staining with PNA (*Peanut Agglutinin*; green); nuclear staining with Hoechst (blue). Scale bar (**A**–**J**) = 7 μm.

To firstly assess the presence and localization of Notch components in bull sperm cells, Notch proteins were analyzed by western blot and immunolocalized using antibodies with known bovine reactivity or here tested for cross-reactivity (Supplementary Figure S1 and Supplementary Figure S2). The western blot analysis demonstrated the presence of all Notch receptors and ligands in ejaculated bull sperm cells (Fig. 1), whereas the immunostaining evidenced that Notch proteins exhibit specific localization patterns (Fig. 2).

NOTCH1 is mainly present in the post-equatorial head segment and weaker in apical ridge and tail (91% of cells; Fig. 2A), whereas NOTCH2 displays three localization patterns, being present in the apical and post-equatorial head segments (64% of cells), or only in the apical region (17%) or in the post-equatorial regions (14%) (Fig. 2B). Additionally, NOTCH2 is detected in the neck region, midpiece and tail of all stained spermatozoa (Fig. 2B). NOTCH3 is present in the equatorial and neck regions, midpiece and tail (93% of cells) or in the post-equatorial head segment (Fig. 2C). NOTCH4 is present in the head equatorial region (70% of cells) or at head base (23%), and in the tail (Fig. 2D).

Regarding Notch ligands, DLL1 is only present in the tail (95% of cells, Fig. 2E), whereas DLL3 is present in the acrosome region, the midpiece and tail (94% of cells, Fig. 2F). DLL4 immunostaining is detected in the acrosome, neck region and midpiece, and a weak signal in the tail (92% of cells, Fig. 2G). JAGGED1 is present in the acrosome apical region (53% of cells), or in both the apical and post-equatorial regions (44%), and in the neck region, midpiece and tail of all sperm cells (Fig. 2H). JAGGED2 is detected in the post-equatorial head region (95% of cells), with a weak signal in the acrosome ridge (Fig. 2I).

### Presence and localization patterns of Notch components in bull sperm cells along the testis, epididymis and *vas deferens*

**Figure 3:**
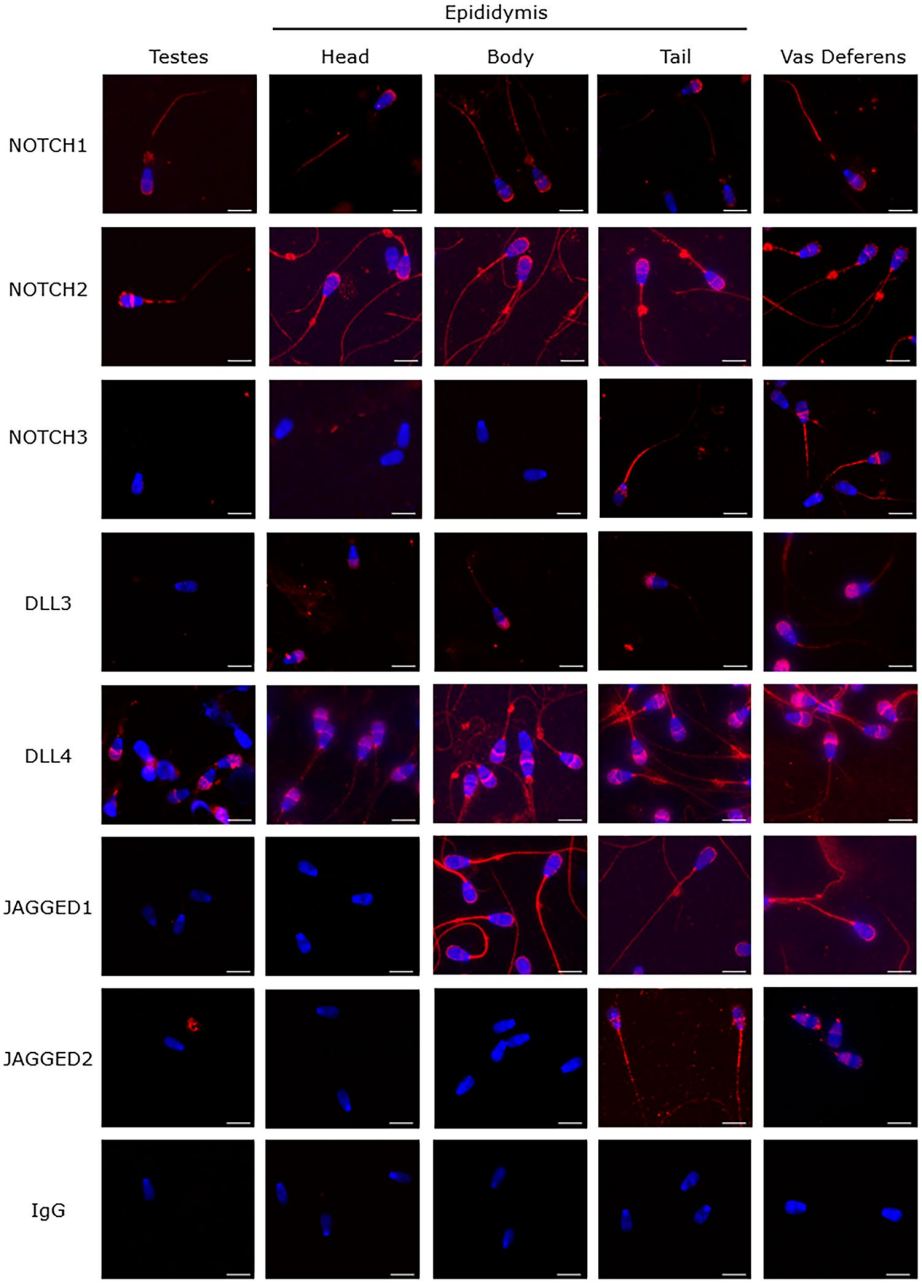
Representative images of NOTCH proteins immunostaining in bull sperm cells recovered along the testes, epididymis and *vas deferens*. Negative control with IgG. Secondary antibody Alexa 594® (red) and nuclear staining with Hoechst (blue). Scale bar = 7 μm.

To evaluate the local origin of Notch components in bull sperm cells, the immunostaining analysis was conducted in spermatozoa recovered from the testes, epididymis and *vas deferens*. This analysis revealed that Notch proteins are acquired by bull sperm cells in a sequential specific pattern (Fig. 3; Table 1). NOTCH1, NOTCH2 and DLL4 are first detected in testicular sperm cells (Fig. 3A). NOTCH1 is detected in the head apical region and tail, NOTCH2 in the head apical and post-equatorial regions, midpiece and tail, and DLL4 in the acrosome and neck regions, midpiece and tail. NOTCH1 and DLL4 maintain this pattern along the epididymis and *vas deferens*, whereas NOTCH2 also acquires a head post-equatorial region localization in the *vas deferens*. In contrast, NOTCH3, DLL3, JAGGED1 and JAGGED2 are first detected along the sequential epididymis segments (Fig. 3B). DLL3 is first detected in sperm acrosome region in the epididymis head and detected in midpiece and tail in the *vas deferens*. JAGGED1 is first detected in sperm apical and post-equatorial head regions, midpiece, neck and tail in the epididymis body. NOTCH3 is first detected in sperm equatorial and/or post-equatorial region, sperm midpiece and tail in the tail epididymis. JAGGED2 is first detected in sperm post-equatorial region in the epididymis tail. DLL1 and NOTCH4 are not detected in pre-ejaculated sperm cells. Overall, these results evidence that sperm NOTCH1, NOTCH2 and DLL4 have a testicular origin, NOTCH3, JAGGED1, JAGGED2 and DLL3 are acquired during epididymal transit, and DLL1 and NOTCH4 are acquired from secretions of accessory glands.

**Table 1:** Detection patterns of NOTCH proteins in bull spermatozoa retrieved from the testis, epididymis (head, corpus, and tail) and *vas deferens*.

Component	Immunolocalization	Epididymis				
		Testis	Head	Body	Tail	Vas deferens
NOTCH1	Head apical	+	+	+	+	+
	Tail	+	+	+	+	+
NOTCH2	Head apical	+	+	+	+	+
	Head post-equatorial	+	+	+	+	+
	Midpiece	+	+	+	+	+
	Tail	+	+	+	+	+
NOTCH3	Equatorial	−	−	−	+	+
	Post-equatorial	−	−	−	+	+
	Midpiece	−	−	−	+	+
	Tail	−	−	−	+	+
DLL3	Acrosome	−	+	+	+	+
	Midpiece	−	−	−	−	+
	Tail	−	−	−	−	+
DLL4	Acrosome	+	+	+	+	+
	Neck	+	+	+	+	+
	Midpiece	+	+	+	+	+
	Tail	+	+	+	+	+
JAGGED1	Head apical	−	−	+	+	+
	Head post-equatorial	−	−	+	+	+
	Neck	−	−	+	+	+
	Midpiece	−	−	+	+	+
	Tail	−	−	+	+	+
JAGGED2	Post-equatorial	−	−	−	+	+

### Relocalization patterns of Notch proteins during sperm acrosome reaction

**Figure 4:**
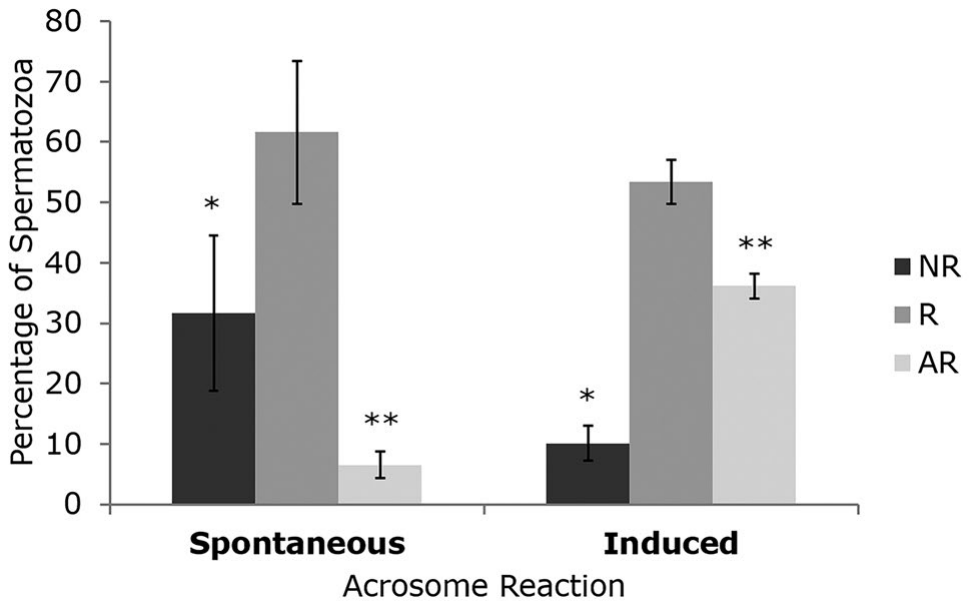
Spontaneous and Calcium (Ca^2+^) Ionophore induced acrosome reaction in bull (*n* = 4) ejaculated sperm cells. *NR* Non-reacted, *R* Reacting, *AR* Acrosome reacted spermatozoa. A minimum of 300 sperm cells were analyzed in each individual sample. The results are represented as mean and the error bars represent standard deviation. *, ** *p* < 0.05 (Fisher exact Test).

Following the detection of the above dynamic localization of Notch proteins, the study proceeded to the evaluation of their potential relocalization during sperm acrosome reaction. This was assessed during both the spontaneous, as well as during the Calcium (Ca^2+^) Ionophore induced acrosome reaction. Acrosome status was assessed using peanut agglutinin (PNA) and classified as follows: (1) non-reacted (NR), representing an intact acrosome with green fluorescence in whole acrosomal cap; (2) reacting (R), indicating a slightly to severely deformed acrosome with a bright or weak fluorescence signal; and (3) acrosome-reacted (AR), representing an acrosome with a fluorescence signal restricted to the acrosomal outline, to the equatorial segment or with no fluorescent signal at all^28^ (Supplementary Figure S3). Induction of acrosome reaction significantly decreased the number of NR sperm and increased the number of AR sperm (*p* < 0.05; Fig. 4).

**Figure 5:**
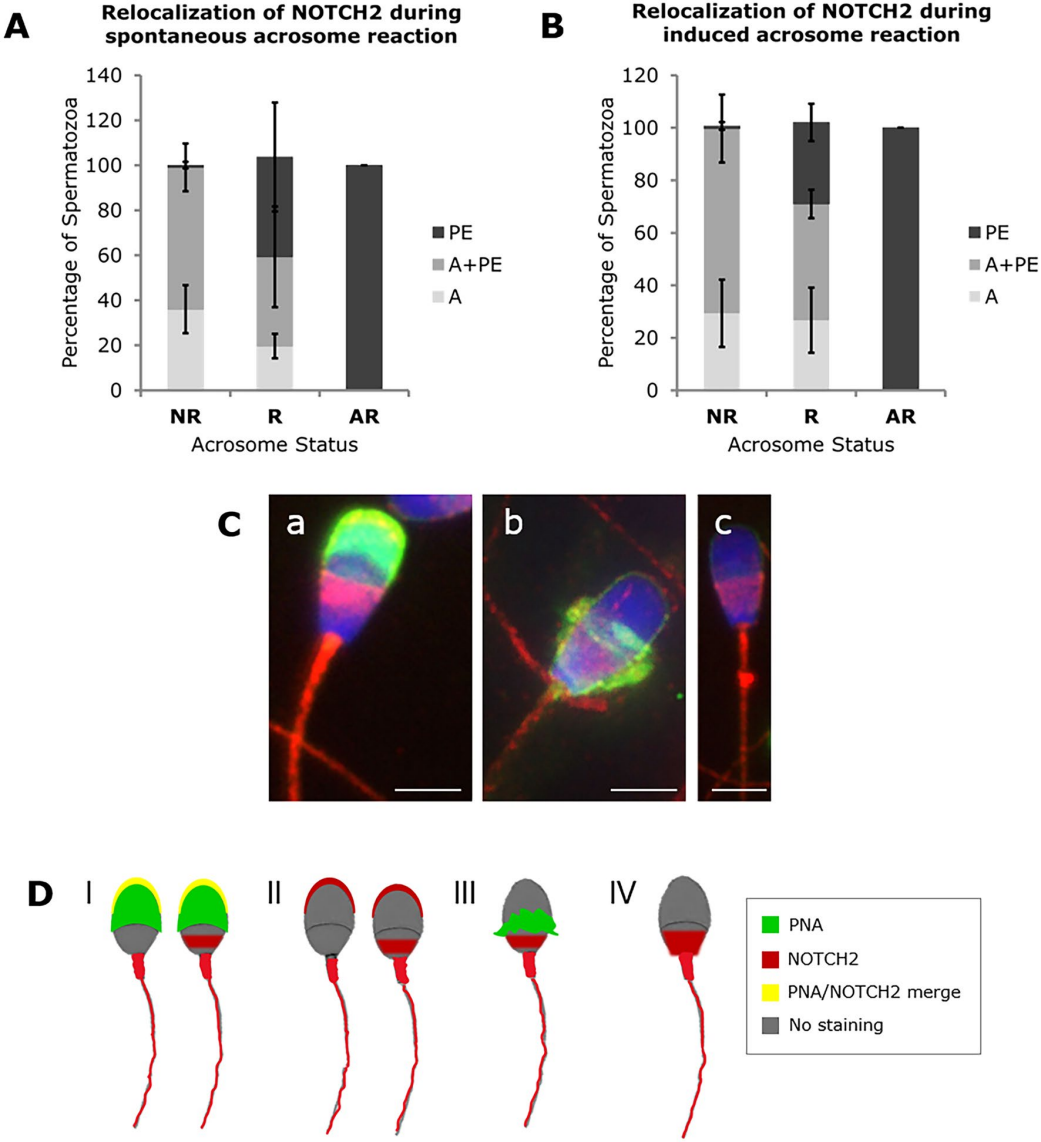
Relocalization patterns of NOTCH2 in bull spermatozoa during spontaneous (**A**) and induced (**B**) acrosome reaction. *NR* Non-reacted, *R* Reacting, *AR* Acrosome reacted spermatozoa, *PE* Head post-equatorial, *Ap* Head apical sperm localization. For both conditions, a minimum of 300 sperm cells were counted in 4 individual samples. The results are represented as means and the error bars denote standard deviation. (**C**) representative images of NOTCH2 immunostaining (red) in NR (a), R (b) and AR (c) sperm cells. Acrosome status stained with PNA and nuclear staining with Hoechst. Scale bar = 4 μm. (**D**) Scheme of NOTCH2 and PNA expression pattern during acrosome reaction. (I) NOTCH2 and PNA merge in NR spermatozoa; (II) NOTCH2 staining in NR spermatozoa with two patterns: apical or apical and post-equatorial head staining; (III) PNA and NOTCH2 staining in reacting spermatozoa; and (IV) NOTCH2 staining in AR spermatozoa with expression in the PE segment, neck, midpiece and tail.

**Figure 6:**
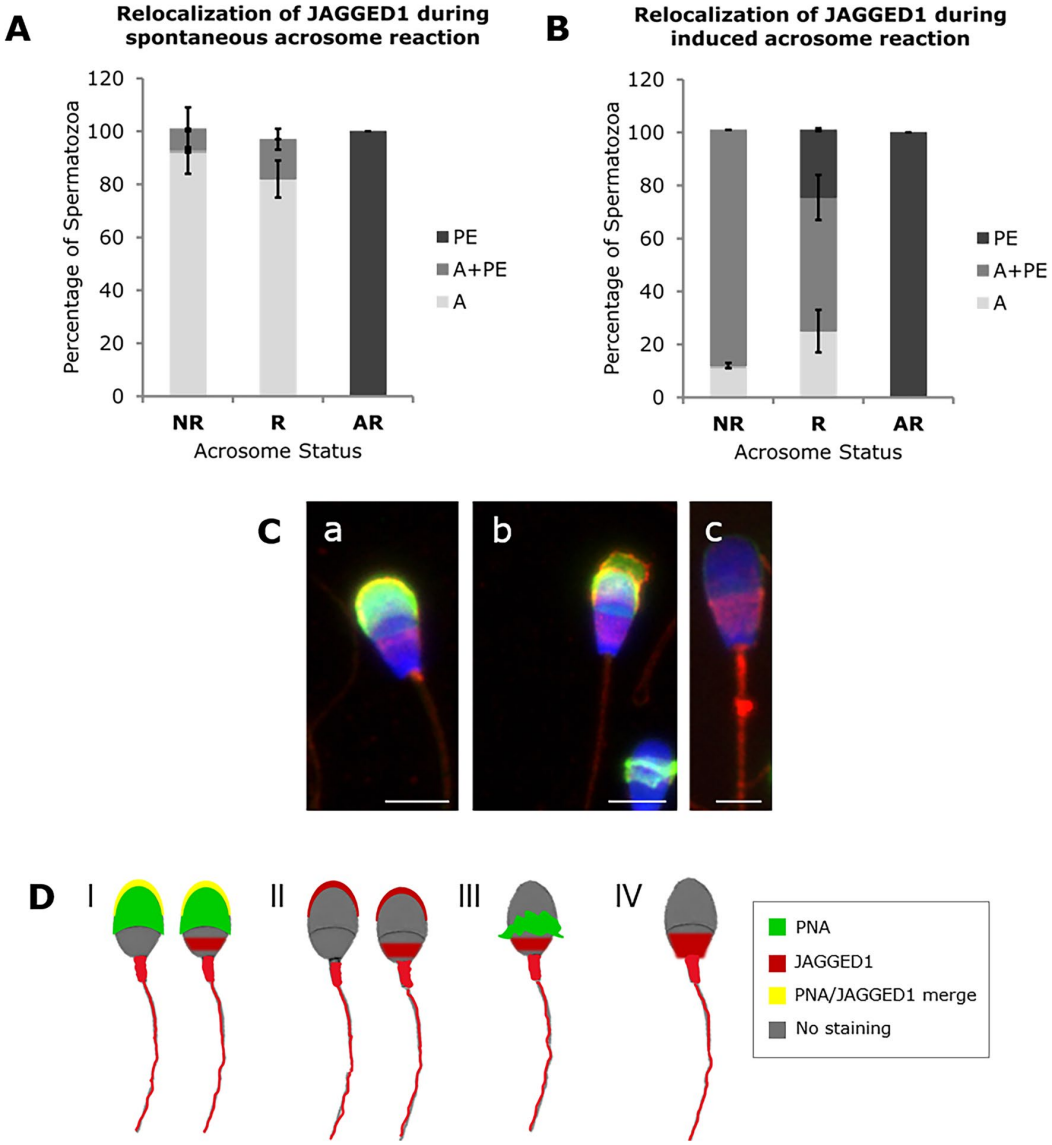
Relocalization patterns of JAGGED1 in bull spermatozoa during spontaneous (**A**) and induced (**B**) acrosome reaction. *NR* Non-reacted, *R* Reacting, *AR* Acrosome reacted spermatozoa, *PE* Head post-equatorial, *Ap* Head apical sperm localization. For both conditions, a minimum of 300 sperm cells were counted in 4 individual samples. The results are represented as means and the error bars denote standard deviation. (**C**) Representative images of JAGGED1 immunostaining (red) in NR (**a**), R (**b**) and AR (**c**) sperm cells. Acrosome status stained with PNA and nuclear staining with Hoechst. Scale bar (a, **b**) = 5 μm; scale bar (**c**) = 3 μm. (**D**) Scheme of JAGGED1 and PNA expression pattern during acrosome reaction. (I) JAGGED1 and PNA merge in NR spermatozoa; (II) JAGGED1 staining in NR spermatozoa with two patterns: apical or apical and post-equatorial head staining; (III) PNA and JAGGED1 staining in reacting spermatozoa; and (IV) JAGGED1 staining pattern in AR spermatozoa with expression in the PE segment, neck, midpiece and tail.

Receptor NOTCH2 and ligands DLL4 and JAGGED 1 exhibit a relocalization pattern associated with sperm acrosome status. As acrosome reaction progresses, NOTCH2 (Fig. 5) and JAGGED 1 (Fig. 6) are relocated from the head apical (Ap) to the post-equatorial (PE) regions, in both spontaneous and induced acrosome reactions. In NR sperm cells, NOTCH2 and JAGGED1 show Ap + PE localization, while AR sperm cells have only a PE detection. The tail detection pattern remains unchanged during acrosome reaction.

**Figure 7:**
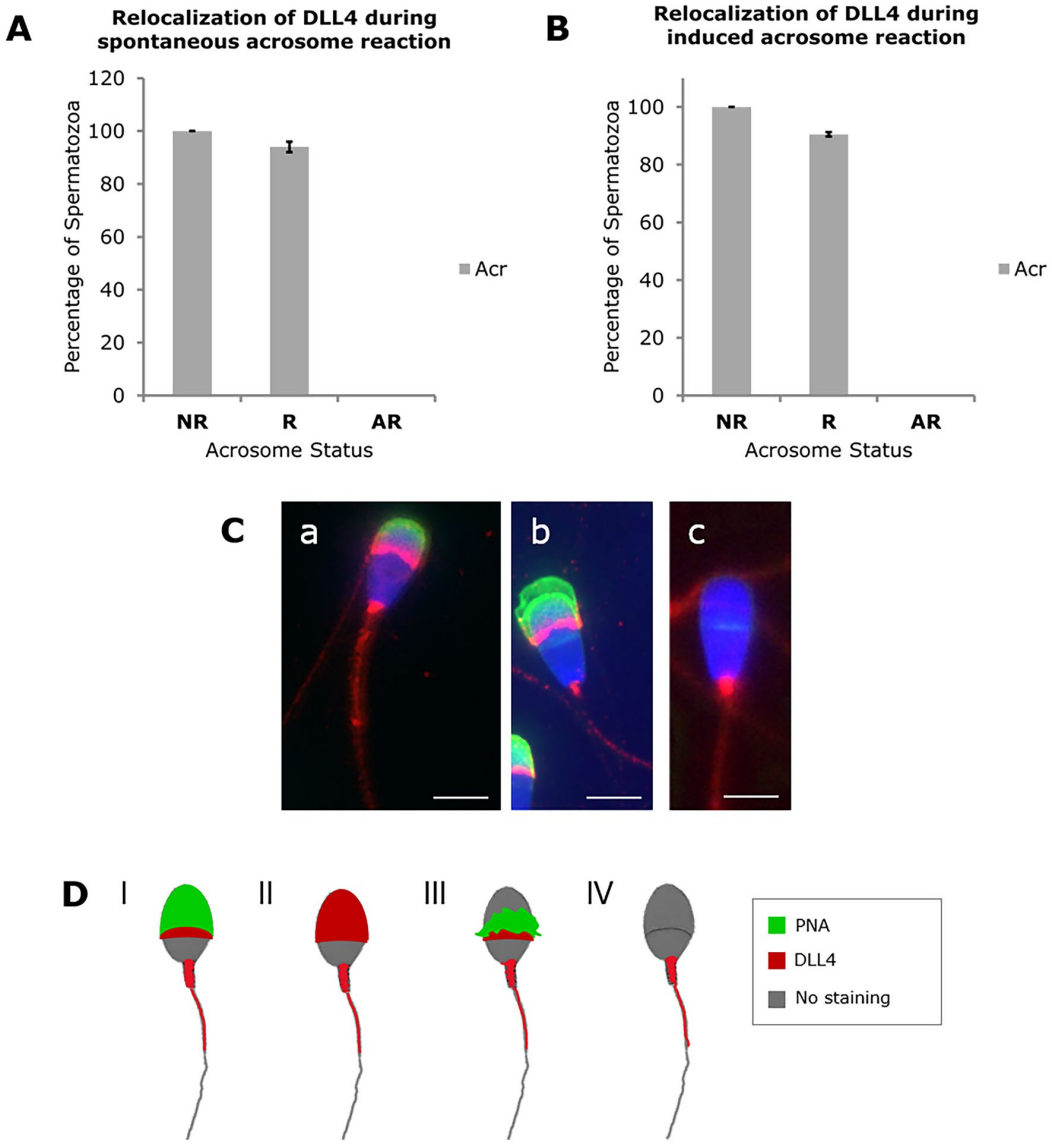
Relocalization patterns of DLL4 in bull spermatozoa during spontaneous (**A**) and induced (**B**) sperm acrosome reaction. *NR* Non-reacted, *R* Reacting, *AR* Acrosome reacted spermatozoa, *Acr* Acrosome. For both conditions, a minimum of 300 sperm cells were counted in 4 individual samples. The results are represented as means and the error bars denote standard deviation. (**C**) Representative images of DLL4 immunostaining (red) in NR (a), R (b) and AR (c) sperm cells. Acrosome status stained with PNA and nuclear staining with Hoechst. Scale bar = 5 μm. (**D**) Scheme of DLL4 and PNA expression pattern during acrosome reaction. (I) PNA and DLL4 in NR spermatozoa. Although DLL4 and PNA share the same location, the two staining do not appear to merge; (II) DLL4 expression pattern in NR spermatozoa with staining in the acrosomal region, neck, midpiece and tail; (III) PNA and DLL4 staining pattern in reacting spermatozoa; (IV) DLL4 staining pattern in AR spermatozoa with no staining in the sperm head and staining in neck, midpiece and tail.

In NR sperm cells, DLL4 is detected in the acrosome, neck region and midpiece. While detection in the neck region and midpiece remains unchanged during acrosome reaction, DLL4 detection in the acrosome region decreases in R sperm, being absent in AR sperm (Fig. 7). These results evidence that the sperm spatial redistribution of NOTCH2 and JAGGED1 is linked to acrosome reaction onset, whereas DLL4 loss is linked to acrosome reaction completion.

### Presence and localization patterns of NOTCH2, DLL4 and JAGGED1 in the testis and epididymis

**Figure 8:**
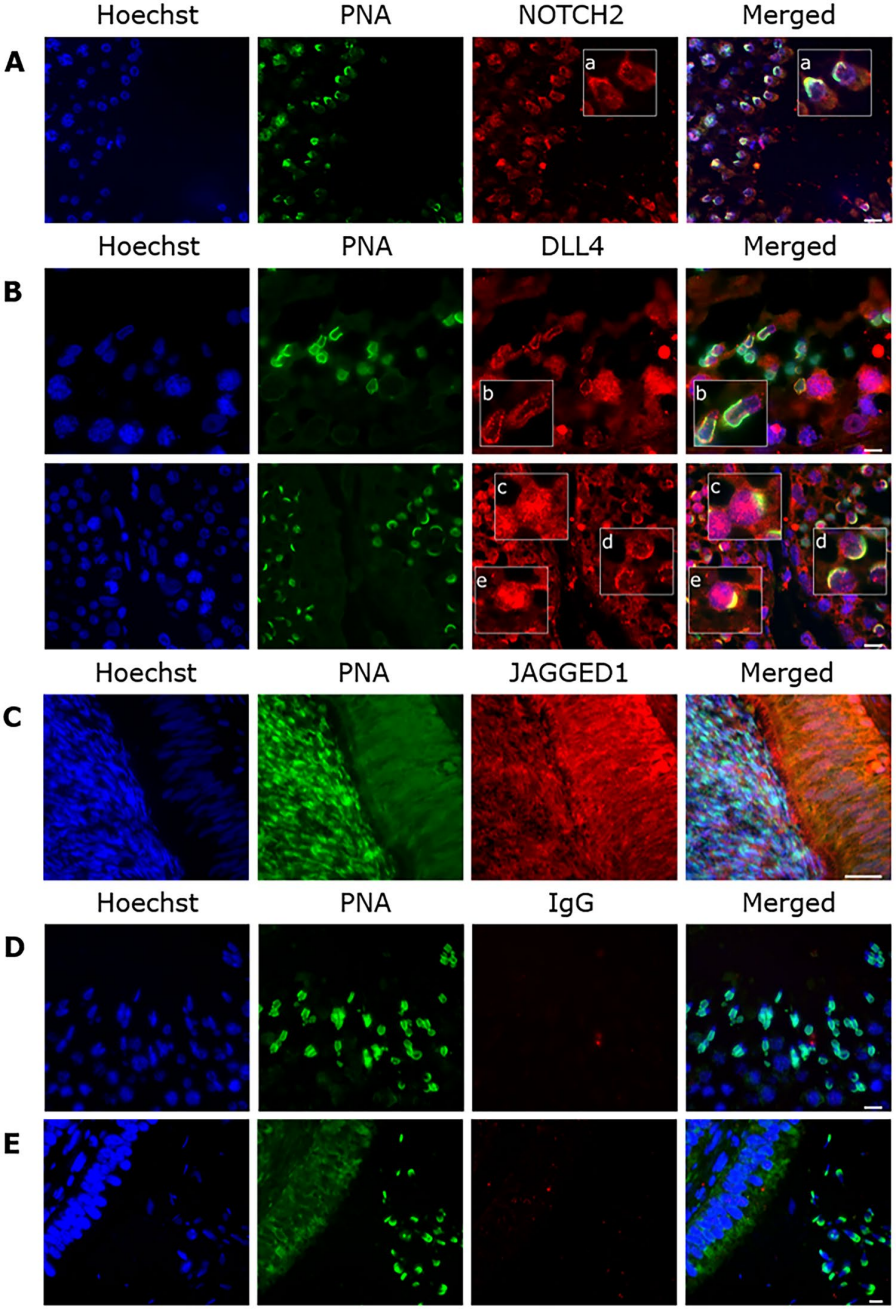
Representative images of expression of Notch components in bull testis and epididymis. (**A**) NOTCH2 expression in the testis, in cytoplasm of germline cells, primarily spermatids in maturation phase (a). (**B**) DLL4 expression the testis in round and elongated spermatids, particularly in the Golgi (b), cap (c), acrosome (d) and maturation (e) phases. (**C**) JAGGED1 expression in the epididymis body epithelium and intraluminal sperm cells. Negative control with IgG in the (**D**) testis and in the (**E**) epididymis. Acrosome staining with PNA (green) and nuclear counterstaining with Hoechst (blue). Scale bar (**A**, **B**, **D**, **E**) = 7 μm; scale bar (**C**) = 3 μm.

Due to the association between NOTCH2, DLL4 and JAGGED1 detection and the course of sperm acrosome reaction, the study proceeded with the evaluation of expression of these Notch components in the testis (NOTCH2, DLL4) and epididymis (JAGGED1). NOTCH2 is detected in the cytoplasm of all germline cells (Fig. 8A), and despite the cytoplasm loss operated in round and elongated spermatids, its expression is kept in the sperm head at the acrosome region (maturation phase). DLL4 is expressed in spermatocytes, round, and elongated spermatids, both in the cytoplasm and in the developing acrosome. Particularly, expression of DLL4 in the developing acrosome of round and elongated spermatids is mainly observed during the Golgi, Cap, Acrosome and Maturation phases (Fig. 8B). In accordance with the detection pattern in intraluminal spermatozoa, JAGGED1 is not expressed in testicular and epididymis head epithelia, being first expressed in the epididymis body epithelium and its intraluminal spermatozoa (Fig. 8C).

## Discussion

This study firstly identified the presence, localization patterns and acquisition origin of Notch proteins in testicular, epididymal, *vas deferens* and ejaculated bull spermatozoa. The study also evidenced for the first time a relationship between the relocation patterns of sperm Notch proteins and the course of sperm acrosome reaction. Finally, the study characterized the sperm-acquisition tissue localization of Notch proteins related to acrosome reaction.

Western Blot analysis results showed that all Notch proteins are detected in bull ejaculated sperm, and immunocytochemistry analysis revealed that all Notch proteins show sperm specific localization patterns. Sperm protein localization is related with specific cellular functions^29^. NOTCH2, DLL4 and JAGGED1 show a strong signal on the apical ridge or the acrosome. Other bull sperm proteins displaying similar detection patterns, such as SPACA1^30^ and P25b^31^ are functionally related to acrosome reaction and sperm-oocyte interaction. NOTCH1-4 and JAGGED1-2 show immunostaining in the sperm head equatorial and/or post-equatorial regions. Proteins located in these regions are often associated with zona-binding and penetration^32^ and sperm-oocyte fusion^33^. NOTCH2-4, DLL1-4 and JAGGED1 exhibit immunostaining in the sperm neck, midpiece and/or tail, protein localizations associated with sperm motility, metabolism, and capacitation^34–36^. As example, bull sperm ADCY10 regulates sperm hyperactivation through a signaling cascade between the sperm neck/midpiece, and the tail^37^, and sperm neck proteins often display immunological functions, probably related to sperm survival in the female genital tract^32^. These results prompt the potential role of Notch proteins in multiple sperm functions. In fact, sperm proteins are known to play critical roles in sperm motility, capacitation, acrosome reaction and fertilization^38^.

Proteins may be added to sperm during spermatogenesis, epididymis/*vas deferens* transit, and following ejaculation through secretions from accessory sexual glands. By recovering sperm cells from these different segments, this study characterized the acquisition origin of sperm Notch proteins. NOTCH1-2 and DLL4 are first detected in testicular spermatozoa, whereas NOTCH3, DLL3 and JAGGED1-2 are first detected during sperm maturation along epididymal transit, and DLL1 and NOTCH4 are only detected in ejaculated sperm, denoting acquisition through secretions of accessory glands^39^.

Sperm maturation is a conserved mammalian event, and epididymal sperm protein binding is well preserved among species^4^. As other sperm proteins^2,29,31^, Notch proteins may also be transferred to spermatozoa through epididymosomes, as evidenced by^26^ in the mouse. In this latter study, JAGGED1 was detected in epididymosomes in different segments of the epididymal lumen. By this process, Notch proteins may be transferred remotely, sequentially contributing to sperm maturation and competence. This concept is in accordance with results of this study, as Notch proteins are acquired in a sequential form along the transit in the epididymis. In fact, DLL3 is first detected in the epididymis head, JAGGED1 in the epididymis body, and NOTCH3 and JAGGED2 in the epididymis tail. Also, detection in different sperm regions is acquired sequentially along sperm transit, as NOTCH3 and DLL3 are first detected in sperm in the epididymis (tail and head respectively) acquiring staining in the sperm midpiece and tail in the *vas deferens*. Protein modifications occurring during sperm transit are often related to sperm tail structural stabilization and increased tyrosine phosphorylation of signaling pathway-related proteins^40^. Acrosome proteins rearranged during epididymal transit may reflect later individual differences among bulls in acrosome stability^30^.

This study evaluated the detection and relocation patterns of Notch proteins during in vitro spontaneous and induced acrosome reaction. Immunofluorescence analysis revealed that NOTCH2, JAGGED1 and DLL4 undergo clear consistent spatial rearrangements during acrosome reaction. NOTCH2 and JAGGED1 are relocated from the head apical to post-equatorial regions, whereas DLL4 is lost along with the acrosome. The immunostaining also allowed the localization of the above proteins on the sperm cell surface, as described for proteins involved in acrosome reaction. Sperm capacitation involves changes in the distribution of sperm membrane proteins^41^, and acrosome reaction results in the fusion of the sperm acrosome and plasma membranes^5^. Upon leaving the testis, sperm plasma membrane contains both membrane-integrated and surface-adsorbed proteins, but during epididymal maturation, surface proteins change their location from one membrane domain to the other^7^, while others are altered, masked, or replaced by new proteins of epididymal origin^8^. Therefore, the sperm surface protein localization of NOTCH2, DLL4 and JAGGED1, and their acrosome status-related relocation patterns during acrosome reaction, points to a relevant role in sperm capacitation. In this context, NOTCH2 and JAGGED1 may be involved in acrosome reaction regulation and DLL4 in acrosome stability. Several bull sperm proteins have already been implicated in sperm-oocyte interactions, such as ADAM1-2^42^, IZUMO1^14^ and E-cadherin^43^, and sperm capacitation results from the interaction of multiple proteins. This requires tiny regulation, and Notch signaling is known for its pivotal role in the regulation of cell-fate and pace of reproductive and developmental events, namely spermatogenesis^19–24,44,45^. In this context, it is noteworthy that all Notch signaling components are present in bull sperm cells. Nevertheless, it's important to highlight that this study used cryopreserved semen, which may have been impacted by cryopreservation-induced changes, such as capacitation-like changes, affecting the sperm protein content and localization patterns. In accordance, Fukuda et al., (2016)^14^ reported that IZUMO1 aberrantly relocalized to whole equatorial segment or whole acrosomal region in bull cryopreserved spermatozoa with damaged acrosomes, a pattern similar to that found in acrosome-reacted spermatozoa. Therefore, we cannot exclude the hypothesis that NOTCH proteins might exhibit a different abundance and/or localization pattern in fresh semen.

As sperm NOTCH2 and DLL4, which are related to acrosome reaction, have testicular origin, this study evaluated their testis cell expression. NOTCH2 is expressed in the cytoplasm of all germline cells, similarly to results described by^25^ in the mouse model. The expression of Notch components in germline cells is linked to spermatogenesis regulation, namely spermatogonia fate^45^, and germline cell fate decisions leading to self-renewal or differentiation^24,25,44,45^. In bull testis, DLL4 is expressed in the cytoplasm of round and elongated spermatids co-localized with the early-stage acrosome development, also similarly to previous findings in the mouse model^25^. The cytoplasmic expression is further confirmed by presence of NOTCH2 and DLL4 in the residual bodies of immature spermatozoa. This points to DLL4 involvement in the regulation of acrosome development, likewise SPACA1, a acrosome membrane protein involved in spermatid acrosome maturation during spermiogenesis^37,46^. Acrosome biogenesis is a crucial phase of spermatid differentiation into a spermatozoon^47^, and Notch signaling was implicated in acrosome formation from the Golgi complex during spermatid maturation^22,44^.

In conclusion, all Notch proteins are present as surface proteins in bull ejaculated sperm. These proteins are already expressed in germ-line cells during spermatogenesis (NOTCH2 and DLL4) or are sequentially acquired by sperm during maturation in epididymal transit (NOTCH3, DLL3 and JAGGED1-2) or through accessory glands following ejaculation (DLL1 and NOTCH4). All these proteins show specific sperm localization patterns, and NOTCH2, DLL4 and JAGGED1 show distinct relocation patterns in the course of spontaneous and induced sperm acrosome reaction. Notch, as major highly conserved signaling pathway involved in regulation of spermatogenesis and other reproductive and developmental events, is by results of this study also prompted for a relevant role in bull sperm physiology and acrosome reaction.

## Methods

### Bull sperm and testicular, epididymal and vas deferens samples

Bull ejaculated sperm samples for the evaluation of the presence and localization patterns of Notch proteins, were selected from cryopreserved semen doses from 4 bulls with proven in vitro and in vivo fertility, stored at the licensed Animal Germplasm Bank of the Reproduction & Development Laboratory. Semen doses were thawed (37 °C, 20 s) and incubated with sperm TALP medium supplemented with pyruvic acid (0.8 mg/mL; P3662, Sigma) and gentamycin (0.1 mg/mL; G1522, Sigma) for 1 h at 39 °C in a 5% CO2 humidified atmosphere. The upper two thirds of the medium column were recovered and centrifuged for 10 min at 200 × g and spermatozoa with high forward motility was recovered from the pellet.

Bull testicular, epididymal and *vas deferens* intraluminal sperm and tissue samples were recovered *postmortem* from 4 mature bulls. Reproductive tracts were collected at a local slaughterhouse, transported at 4 °C within 3 h to the laboratory, cleaned with ethanol 70%, and the testes, epididymis and *vas deferens* individualized. To recover testicular sperm, testes were dissected, tissue infused with 5 mL of saline and gently squeezed, and spermatozoa enriched fluid recovered. To recover epididymal sperm, the epididymis was first divided in its three segments (head, body and tail). Epididymis head spermatozoa were recovered as testicular sperm, whereas epididymis body and tail, and *vas deferens* spermatozoa were recovered using an intraluminal flushing technique. Recovered intraluminal fluids were centrifuged for 1 min at 300 × g at RT, the supernatant collected and centrifuged for 10 min at 300 × g, and the sperm pellet washed twice in PBS.

To obtain tissue samples, testes, epididymis and *vas deferens* were cut into sections, fixed in 10% formalin for 24–48 h at room temperature, embedded in paraffin, and 3 μm thick tissue sections used for immunohistochemistry.

### Bull ejaculated sperm and tissue protein extraction and SDS-PAGE immunoblotting (Western Blot)

Following swim-up, sperm cell suspensions were centrifuged for 10 min at 400 × g, and 30 × 10^6^ sperm cells lysed in RIPA buffer (RIPA Buffer, cat. 89,901, Thermo Scientific) supplemented with protease inhibitors (A32953, Pierce™, Thermo Scientific) for 30 min in ice, under gentle stirring. Suspensions were then centrifuged (10 min, 16,000 × g, 4 °C) to recover the supernatant (soluble protein fraction), to quantify total protein concentrations using the Bradford method (MB19801, Nzytech). To extract tissue proteins (mouse and bovine liver and heart, from a tissue bank), 20 mg of each tissue was lysed as above, plus a disruption step using TissueLyser (3 cycles of 25HZ, 30 s each), and protein recovery and quantification performed as described above. For SDS-PAGE, 60 µg of total protein was boiled with Laemmli Buffer (M11701, Nzytech), samples run on 4% stacking and 10% separating polyacrylamide gel (Bio-Rad electrophoresis system), and proteins transferred to a PVDF membrane (LC2007, Invitrolon™/Invitrogen™). After blocking with 5% skim milk and 0.05% Tween-20, membranes were incubated with the primary antibodies overnight at 4 °C and incubated with the secondary antibody HRP labeled (Table 1) for 1 h at RT, being the signal detected by the Super Signal™ chemiluminescence substrate (34,580, Thermo Scientific). DLL1 validation western blot the chemiluminescent signal was detected via the use of X-ray films. Otherwise, a digital detection system was use (Chemidoc Image Lab Software XRS + , Bio-Rad).

### Immunolocalization of Notch components in bull ejaculated sperm

Following swim-up, motile spermatozoa were cytospin centrifuged (4 min at 1500 rpm), dropped on a slide and air dried for 10 min. After fixation with 1% PFA (paraformaldehyde, 8.18715, Sigma) for 30 min at 4 °C, slides were washed twice with PBS 1 × for 5 min and blocked with 2.5% BSA (bovine serum albumin, A7906, Sigma) and 0.05% Tween-20 (EC-607, National Diagnostics, USA) for 1 h in a humidified dark chamber at room temperature (RT). Slides were then incubated with the primary antibodies (Supplementary Table S4) overnight at 4 °C. The Rabbit control IgG (Supplementary Table S4) was used as a negative control. After washing with PBS 1x, slides were incubated with the secondary antibody (Alexa Fluor™ 594 goat anti-rabbit, ab150080, Abcam) for 30 min at RT, washed and incubated with Peanut Agglutinin (3 µg/mL; Lectin PNA Alexa Fluor™ 488, L21409, Invitrogen™) for 15 min to evaluate acrosome status (non-reacted—NR, reacting—R, acrosome reacted—AR). Slides were then washed and incubated with bisBenzimide H 33,258 (1 µg/mL; b2883, Sigma) for 10 min for nuclear staining, again washed, and mounted with ProLong™Gold antifade mounting medium (P36934, Invitrogen™). Images were acquired in an inverted epifluorescence microscope (Leica, DMR), and analyzed and treated with Adobe Photoshop (CS5 21.1.1). For each Notch protein, 8 fields were acquired per slide and 900 spermatozoa counted.

### In vitro sperm capacitation and acrosome reaction

Following swim-up, sperm cells were incubated in TALP medium supplemented with heparin (60 µg/mL; H3393, Sigma), and incubated for 3 h at 39 °C in 5% CO2 for in vitro capacitation. Capacitated sperm cells were then centrifuged at 200 × g for 10 min, the pellet resuspended in TALP medium and divided into two aliquots (2 × 10^6^ spermatozoa/mL). The first aliquot was left untreated to evaluate the spontaneous acrosome reaction, while the second aliquot was incubated with Ca^2+^ ionophore (0.7 µM; C7522, Sigma) to evaluate the induced acrosome reaction. Both aliquots were then incubated for 1 h at 39 °C in 5% CO2, processed for Immunocytochemistry as described above, using PNA lectin and the anti-Notch primary antibodies. A minimum of 300 spermatozoa per Notch protein and bull were analyzed.

### Tissue sections immunohistochemistry (IHC)

Tissue slides were deparaffinized by heating at 56 °C for 5 min and incubation in xylol for 20 min, and rehydrated in serial ethanol solutions (100%, 95%, 70% and H2O final step). Antigen retrieval was performed in a microwave (3 × 5 min) in citrate buffer (10 mM; pH 6.0), and the thereafter blocking step and the remaining IHC protocol, as well as image acquisition, performed as described above.

### Ethics approval and consent to participate

Not applicable.

### Supplementary Information

Supplementary Information.

## Data Availability

All data supporting the findings of this study are included in this paper and its additional files.
