# Supplementary material for: Characterization of expression patterns and dynamic relocation of Notch proteins during acrosome reaction of bull spermatozoa
Source: Sci Rep. 2024 Jun 28;14:14925. doi: 10.1038/s41598-024-65950-0 (PMC11213903; doi:10.1038/s41598-024-65950-0)
Supplement: Supplementary file 1 — Supplementary Information. [file 41598_2024_65950_MOESM1_ESM.pdf]

Characterization of expression patterns and dynamic relocation of Notch proteins during acrosome reaction of bull spermatozoa

Patrícia Diniz[1,2*] Inês Leites[1,2*], Mariana Batista[1,2,3], Ana Catarina Torres[1,2], Luísa Mateus[1,2], Luís Lopes-da-Costa[1,2], Elisabete Silva[1,2 †]

<table>
<tr><th>Western Blot</th><th>Bands considered</th></tr>
<tr><td>
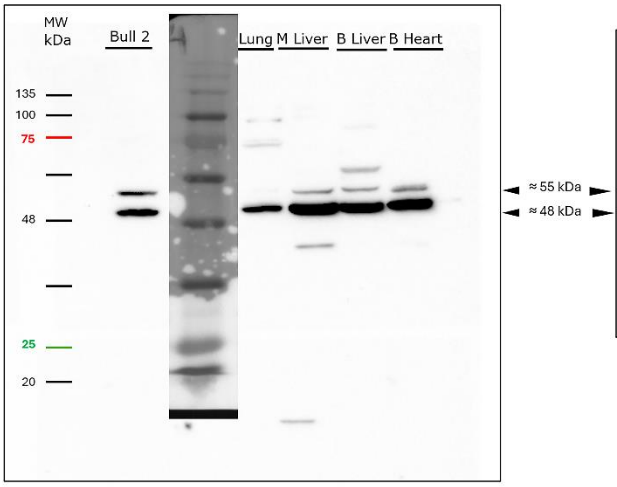

Exposure 300.0sec
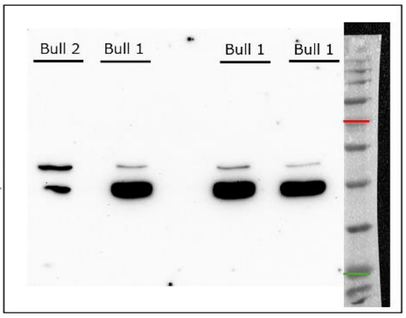

Exposure 300.0sec
</td><td>Anti-NOTCH4 antibody detects the expected protein isoforms of 55 and 48 kDa (datasheet).
The sperm sample from bull 2 and the target tissues were run in one gel; while the sperm sample from bull 1 and a replicate from bull 2 were run in a different gel.</td></tr>
<tr><td>
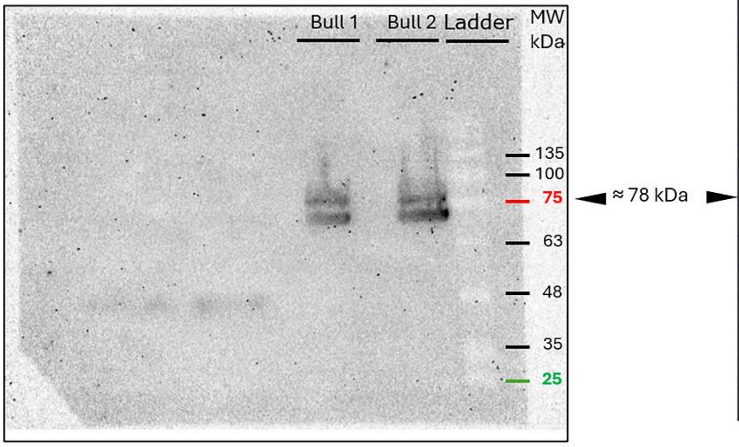

Exposure 30.0sec
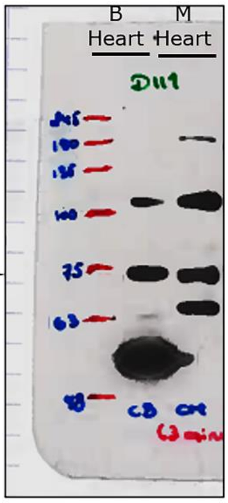

</td><td>DLL1 was detected in target tissues and spermatozoa at 78 kDa, as predicted in the antibody datasheet.
Samples from bulls 1-2 and samples from target tissues were run in different gels.</td></tr>
<tr><td>
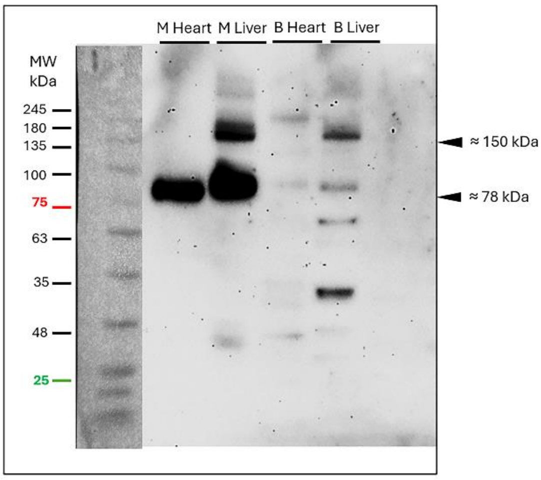

Exposure 900.0sec
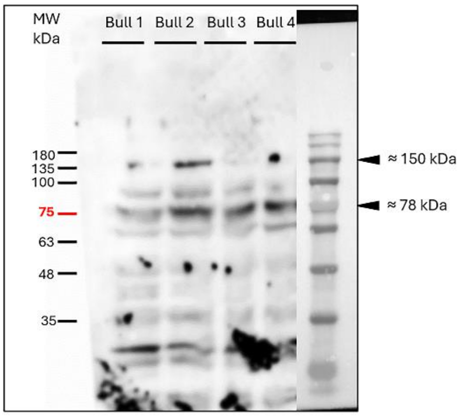

Exposure 396.4sec
</td><td>JAGGED2 predicted weight is 129-133 kDa (datasheet). The observed weight in bull spermatozoa is around 150 kDa. An additional band with a higher expression was detected at 78 kDa, which may correspond to a protein isoform.
Samples from tissues and spermatozoa were run in different gels.</td></tr>
</table>

Supplementary Figure S1. **Antibody validation by Western Blot.** SDS-PAGE of proteins extracted from bovine spermatozoa (n=4 bulls) and mouse (M) and bovine (B) tissues. Western Blots were performed for antibodies with no described cross-reactivity/predicted reactivity with bovine species. In cases where multiple bands were detected, the band with the predicted weight, as described in the antibody datasheet, was considered.

Notch component	Western Blot	Bands considered	Ref.
NOTCH1	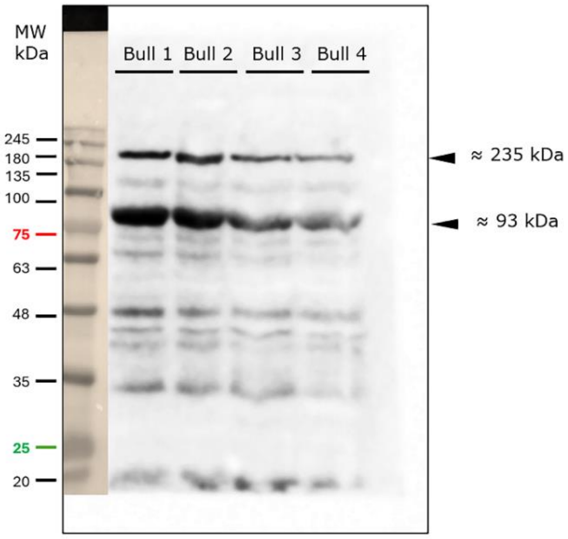 Exposure 30.0sec	NOTCH1 was detected in spermatozoa as predicted at 93 kDa, corresponding to the active form, and at 235 kDa corresponding to the full-length protein. Samples were run in the same gel.	[1]
NOTCH2	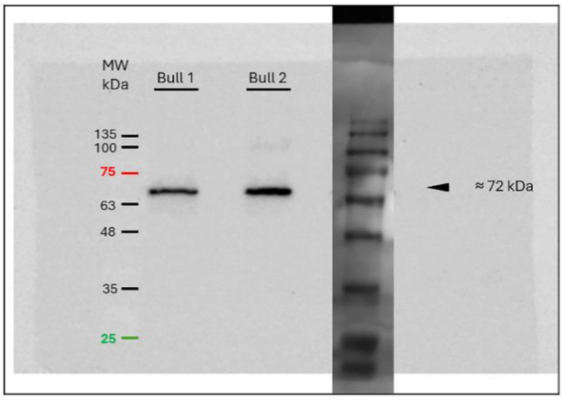 Exposure 300.0sec 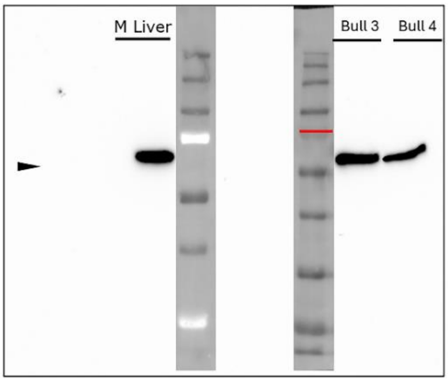 Exposure 300.0sec	Anti-NOTCH2 antibody detects the full-length protein, cleaved forms, post-translational modified forms and the NICD (Notch Intracellular Domain). In bull spermatozoa, the protein was detected at 72 kDa possibly corresponding to NOTCH2ICD, as previously described. Samples from bulls 1-2, and 3-4 were run in different gels.	[2]

NOTCH3	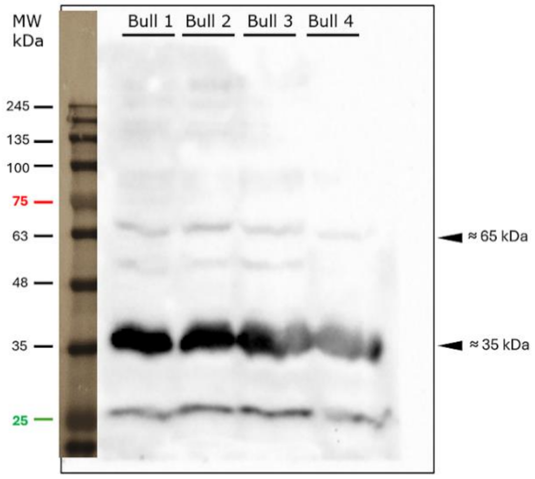<br>Exposure 60.0sec	NOTCH3 predicted weight is 280 and 120 kDa. In bull spermatozoa, it was detected with a higher expression at around 35 kDa.<br>Samples were run in the same gel.	
DLL3	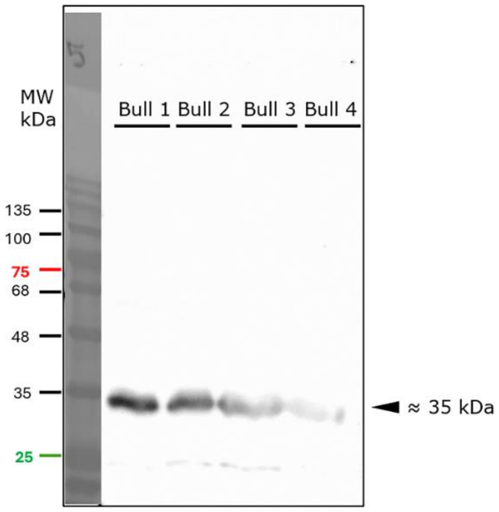<br>Exposure 600.0sec	DLL3 predicted weight is 63 kDa. In bull spermatozoa, it was detected as a single band at 35 kDa.<br>Samples were run in the same gel.	
DLL4	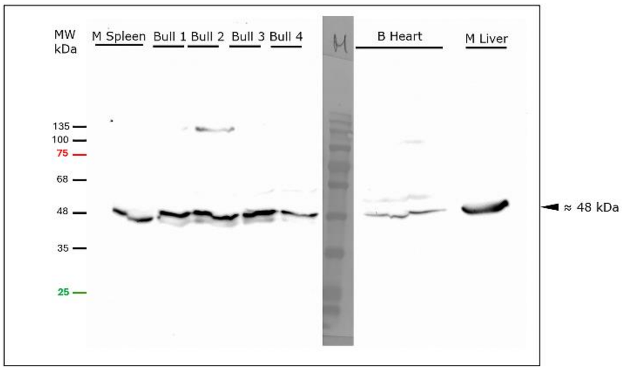<br>Exposure 1500.0sec	DLL4 predicted weight is 74 kDa. In bull spermatozoa, it was detected as a single band at 48 kDa.<br>Samples were run in the same gel.	

JAGGED1	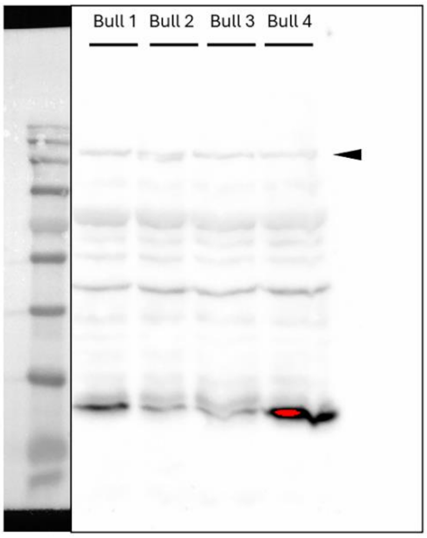<br>Exposure 30.0sec<br>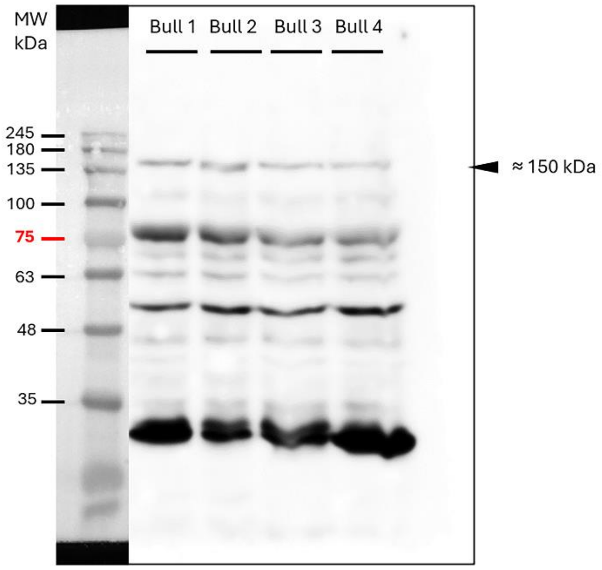<br>Exposure 60.0sec	Anti-JAGGED1 antibody detects the full-length protein at 150 kDa which was detected in bull spermatozoa. Other bands with a high expression (78, 50 and 35 kDa) should correspond to protein isoforms. Samples were run in the same gel.	

Supplementary Figure S2. Representative full-length Western Blots showing Notch proteins in bull cryopreserved spermatozoa. In cases where multiple bands were detected, the band with the predicted weight, as described in the antibody datasheet, was considered. Otherwise, the band(s) with more expression were considered. For JAGGED1 more than one exposure is displayed.

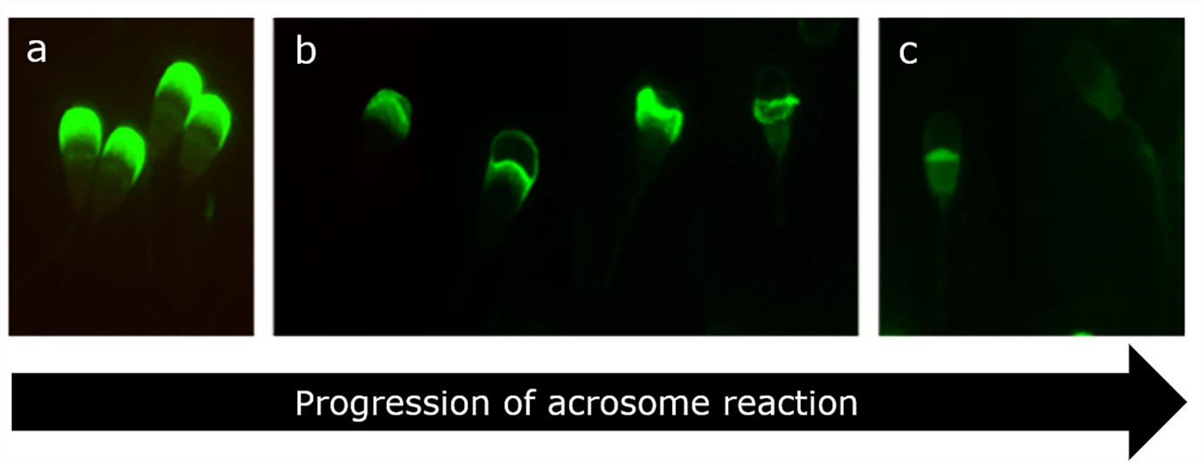

Supplementary Figure S3. Classification of bull spermatozoa according to acrosome status assessed with PNA (Peanut Agglutinin). (a) Non-reacted (NR) spermatozoa; (b) reacting (R) spermatozoa; (c) Acrosome reacted (AR) spermatozoa.

Antibody	Type	Host	Isotype	Application / Dilution	Source	Cross-reactivity
NOTCH 1	Primary	Rabbit polyclonal	IgG	IHC/ICC (1:100) WB (1:500)	ab8925 (Abcam)	[3]
NOTCH 2	Primary	Rabbit polyclonal	IgG	IHC/ICC (1:200) WB (1:5000)	ab8926 (Abcam)	[4]
NOTCH 3	Primary	Rabbit polyclonal	IgG	IHC/ICC (1:50) WB (1:500)	sc-5593 (Santa Cruz Biotechnology)	[5]
NOTCH 4	Primary	Rabbit polyclonal	IgG	IHC/ICC (1:100) WB (1:500)	sc-5594 (Santa Cruz Biotechnology)	Validated for this study (Supplementary Fig. S1)
DLL1	Primary	Rabbit polyclonal	IgG	IHC/ICC (1:200) WB (1μg/mL)	ab76655 (Abcam)	Validated for this study (Supplementary Fig. S1)
DLL3	Primary	Rabbit polyclonal	IgG	IHC/ICC (1:50) WB (1:500)	sc-67270 (Santa Cruz Biotechnology)	Cross-reactivity
DLL4	Primary	Rabbit polyclonal	IgG	IHC/ICC (1:100) WB (1:10000)	DF13221 (AffiBiotech)	Predicted cross-reactivity
JAGGED1	Primary	Rabbit polyclonal	IgG	IHC/ICC (1:50) WB (1:500)	sc-8303 (Santa Cruz Biotechnology)	Cross-reactivity
JAGGED2	Primary	Rabbit polyclonal	IgG	IHC/ICC (1:100) WB (1:1000)	144-63149-50 (RayBiotech)	Validated for this study (Supplementary Fig. S1)
IgG	Primary	Rabbit polyclonal	IgG	N/A	ab27478 (Abcam)	N/A
AlexaFluor® 594 goat anti-rabbit	Secondary	Goat	IgG	IHC/ICC (1:300)	ab150080 (Abcam)	N/A
Goat anti-rabbit HRP	Secondary	Goat	IgG	WB (1:10000)	P0448 (Dako)	N/A

Supplementary Table S4. Antibody panel. List of antibodies used in the study for IHC (immunohistochemistry), ICC (immunocytochemistry) and WB (western blot). For antibodies with no cross-reactivity with bovine, a homology search of antibody sequences was performed using BLAST (Basic Local Alignment Tool https://blast.ncbi.nlm.nih.gov/Blast.cgi). Antibody validation was performed by Western Blot.

References

1. MacKenzie, M. G., Hamilton, D. L., Pepin, M., Patton, A. & Baar, K. Inhibition of Myostatin Signaling through Notch Activation following Acute Resistance Exercise. PLoS ONE 8, e68743 (2013).

2. Sharma, M. et al. Activation of Notch signaling pathway in HIV-associated nephropathy. AIDS 24, 2161–2170 (2010).

3. Marsolier, J. *et al.* Theileria parasites secrete a prolyl isomerase to maintain host leukocyte transformation. *Nature* **520**, 378–382 (2015).

4. Dang, W. *et al.* Notch2 Regulates the Function of Bovine Follicular Granulosa Cells via the Wnt2/β-Catenin Signaling Pathway. *Animals* **14**, 1001 (2024).

5. Bonadeo, N., Becu-Villalobos, D., Cristina, C. & Lacau-Mengido, I. M. The Notch system during pubertal development of the bovine mammary gland. *Sci. Rep.* **9**, 8899 (2019).
